# Turning Slowly Predicts Future Diagnosis of Parkinson's Disease: A Decade‐Long Longitudinal Analysis

**DOI:** 10.1002/ana.78034

**Published:** 2025-10-21

**Authors:** Morad Elshehabi, Clint Hansen, Markus A. Hobert, Anna‐Katharina von Thaler, Kathrin Brockmann, Bhargav Tallapragada, Florian Metzger, Daniela Berg, Walter Maetzler, Brook Galna

**Affiliations:** ^1^ Department of Neurology University Medical Center Schleswig‐Holstein and Kiel University Kiel Germany; ^2^ Center for Neurology University Hospital Tübingen and Hertie Institute for Clinical Brain Research Tübingen Germany; ^3^ Department of Neurology University Medical Center Schleswig‐Holstein and University of Lübeck Lübeck Germany; ^4^ Neurodegenerative Diseases University Hospital Tübingen, and Center for Neurodegenerative Diseases Tübingen Germany; ^5^ School of Psychology Murdoch University Perth Australia; ^6^ School of Allied Health (Exercise Science) | Centre for Healthy Ageing | Personalised Medicine Centre Murdoch University Perth Australia; ^7^ Geriatric Center and the Department of Psychiatry and Psychotherapy University Hospital Tübingen Tübingen Germany

## Abstract

**Objective:**

Wearable technology allows accurate measurement of turning while walking, with cross‐sectional studies indicating that difficulty turning presents even in preclinical phases of Parkinson's disease. The aim of our study was to quantify rate of change of turning performance in a cohort of older adults, and test whether turning decline can predict future diagnosis of Parkinson's disease.

**Methods:**

A total of 1,051 participants from the Tübingen Evaluation of Risk Factors for Early Detection of Neurodegeneration (TREND) study were included for a 5‐visit analysis over 10 years, with development of clinically evident Parkinson's disease tracked. Participants walked a 20‐m hallway for 1 minute at their preferred pace, with a wearable device on the lower back. Longitudinal trajectories of turning performance were modelled using random effects linear mixed models to establish the interval between initial turning changes and Parkinson's disease diagnosis. Cox regression assessed whether initial turning measures could predict time to Parkinson's disease onset, controlling for age and sex.

**Results:**

Of all participants, 23 were diagnosed with Parkinson's disease an average of 5.3 years post‐baseline. Slower peak angular velocity at baseline was associated with a higher hazard of Parkinson's disease diagnosis, with deviations from controls emerging approximately 8.8 years before diagnosis. Additional analysis with a machine learning model using baseline characteristics of age, sex and peak angular velocity, identified 60% of prediagnostic Parkinson's disease (sensitivity: 0.600) and 80.5% non‐prediagnostic Parkinson's disease (specificity: 0.805), with an area under the curve of 80.5%.

**Interpretation:**

Peak angular velocity during turning shows promise identifying and tracking motor progression in the pre‐diagnostic phase of Parkinson's disease. ANN NEUROL 2026;99:114–123

Parkinson's disease (PD) is a neurodegenerative disorder characterized by a progressive course, including an early prediagnostic (also commonly known as prodromal) phase during which the disease is present, but its symptoms are insufficiently pronounced to allow a clinical diagnosis.^1^, ^2^, ^3^ Detecting individuals in this early stage is of considerable importance, because it represents the window during which neuroprotective treatments should ideally be administered once available. Hence, many studies have aimed to define the prediagnostic period of PD in terms of its features and duration.^3^ Estimates from both retrospective and prospective studies suggest that this prediagnostic phase spans between 3 and 15 years.^4^, ^5^

Research into non‐motor markers for PD has yielded promising results. For example, Kluge et al^6^ found that participants of the Tübingen Evaluation of Risk Factors for Early Detection of Neurodegeneration (TREND) study later diagnosed with clinically diagnosed PD showed positive α‐synuclein seed amplification assay (SAA) in blood 1 to 10 years before clinical diagnosis. Constipation and speech difficulties have also been shown to yield predictive potential in prediagnostic PD (PPD).^7^ Darweesh et al^8^ showed a decline in preclinical PD compared to controls in instrumented daily life activities, movement poverty, bradykinesia, tremor, and cognitive abilities, up to 7 years before the clinical diagnosis.

Moreover, findings in mobility markers have been increasingly encouraging.^9^ Schalkamp et al^10^ investigated 103,712 participants from the United Kingdom (UK) Biobank and showed that a decline in wrist acceleration during light physical activity could predict PD diagnosis in 153 participants approximately 7 years earlier. Our own work with longitudinal data from the TREND cohort^11^ showed changes in straight walking measures approximately 4.5 years before a clinical PD diagnosis was possible. Namely, gait variability and asymmetry were the most promising measures in this regard.

Turning is a complex part of walking. It requires constant coordination, re‐orientation, and modification of the locomotor trajectory while moving.^12^ Difficulties turning are common and highly relevant to daily life in PD.^12^, ^13^ Therefore, it seems intuitive that turning deficits could be observed in PPD,^13^ emphasizing the need to investigate the predictive potential of turning measures in this phase.

Wearable digital devices for gait analysis have shown promise over the past 2 decades.^14^, ^15^ These devices offer objective, granular quantification of measures that are difficult to assess solely through clinical observation.

The aims of this longitudinal prospective observational study over a period of 10 years in a cohort of older adults with and without (non‐motor) risk markers for PD recruited from the community^16^ were to: (1) quantify longitudinal trajectories of turning performance and test whether these trajectories change differently in people who were and were not subsequently diagnosed with PD; (2) investigate if baseline turning performance was associated with a higher hazard of PD, and, if yes; (3) identify the time at which changes in turning emerge before PD diagnosis. We hypothesized the following: (1) turning worsens more quickly in PPD compared to non‐PPD; (2) worse turning performance is associated with a higher hazard of PD; and (3) turning performance diverges between controls and PPD years prior PD diagnosis, as we have previously reported for straight walking.^11^

## Methods

The longitudinal data included in this analysis were collected over 5 visits between 2013 and 2023 (T1–T5, because of the coronavirus disease 2019 pandemic, T5 required a longer duration and had a reduced number of participants). A total of 1,051 participants from the TREND study were included.

The TREND study was conducted at the Departments of Neurology and Psychiatry, University Hospital Tübingen, Germany, in accordance with the Declaration of Helsinki, with ethical approval from the Ethics Committee of the Medical Faculty, University of Tübingen (no. 90/2009BO2). All participants gave their written informed consent before testing.

### Demographic and Clinical Measures

For each participant, age, sex, body mass index, years of education (as defined by the International Standard Classification of Education [ISCED]),^11^ and Beck Depression Inventory I (BDI‐I) score,^17^ and self‐reported depression^18^ were recorded. The assessment battery also included the Movement Disorder Society's Unified Parkinson's Disease Rating Scale part III (MDS‐UPDRS‐III), the Mini‐Mental State Examination (MMSE), and Consortium to Establish a Registry for Alzheimer's Disease (CERAD).^19^ Inclusion criteria for the TREND study required participants to be adults over the age of 50, without significant hearing or visual impairments. Exclusion criteria were a diagnosis of neurodegenerative disease, stroke, inflammatory central nervous system disease, or the use of dopaminergic or antipsychotic medications, as well as any cardiothoracic or orthopedic conditions that could notably impair walking during testing.

The Prodromal Parkinson's Disease Probability Score was calculated for each study visit based on the method described in the Movement Disorders Society (MDS) research criteria for prodromal PD.^2^ Sleep behavior disorder (RBD) assessed using the RBD Screening Questionnaire,^20^ hyposmia measured by the Sniffin’ Sticks test battery^21^ using its normative values,^22^ and a self‐reported history of acute presence of major depressive symptoms^18^ was recorded. Symptomatic orthostatic hypotension, urinary dysfunction, erectile dysfunction, and constipation, were all evaluated using the Unified Multiple System Atrophy Rating Scale (UMSARS).^23^

### Laboratory Data Collection: Equipment and Gait Protocol

Each participant wore multiple Opal sensors (APDM, Portland, OR, 128 Hz) with 1 positioned on the lower back at the level of the fourth and fifth lumbar vertebrae (L4, L5). All participants completed a 1‐minute walking trial at a normal pace along a 20‐m hallway.^24^ Turning characteristics were calculated from 3 or more turns for over 90% assessments of assessments meeting this criterion. Seven turning measures derived from the accelerometer and gyroscope signals were analyzed with a validated algorithm, as described in Pham et al.^25^ Namely, we derived and analyzed turn angle, turn duration, as well as angular velocity at the beginning, middle, and end of the turn, along with the average and peak angular velocity over the whole turn. In the validation study,^25^ turn angle was not among the validated measures. Therefore, to ensure reliability, we refrained from drawing conclusions based on this measure.

### Statistical Analysis

Statistical analysis was conducted using R statistics,^26^ specifically the “lme4”^27^ and “survival”^28^ packages.

#### Changes in Turning Measures Overtime

We modelled annual change in turning performance using linear mixed models. Random intercepts and slopes (annual change) were included for each participant, while PPD status, sex and age were treated as fixed effects. To compare turning measures between the PPD cohort and controls, we centered the age of the PPD group at the time of PD diagnosis, and the age of the control group was centered at the mean age of diagnosis of the PPD group (77.1 years).

#### Survival Analysis

Cox proportional hazards regression was used to assess whether turning measures predicted PD diagnosis, specifically whether turning performance was associated with a higher hazard of receiving a PD diagnosis. First, a base model was constructed including demographic and clinical variables: baseline age, sex, body mass index, global cognition (MMSE), and depressive symptoms (BDI). Next, turning measures were individually added to the base model. The classification performance for significant survival models was estimated using extreme gradient boosting (“XGBoost” package for R statistics), where the dataset was divided into 75% training and 25% testing sets and the frequency of PPD up‐sampled to militate against classification bias toward the predominant class (non‐PPD). Classification model hyperparameters were tuned using 1,000 bootstrap resamples of the training data and the final model was evaluated on the naïve test set.

#### When Does Turning Start to Change before the Clinical PD Diagnosis?

We estimated how many years before PD diagnosis turning began to diverge from controls using established methods.^11^, ^29^ This involved calculating the difference between PPD and controls at the time of diagnosis, then dividing this between‐group difference by the yearly change observed in the PPD group. This analysis was conducted for all turning measures associated with a higher hazard of developing PD and that significantly changed over time in the PPD group.

## Results

### Demographics and Wearable Device Data

**FIGURE A1:**
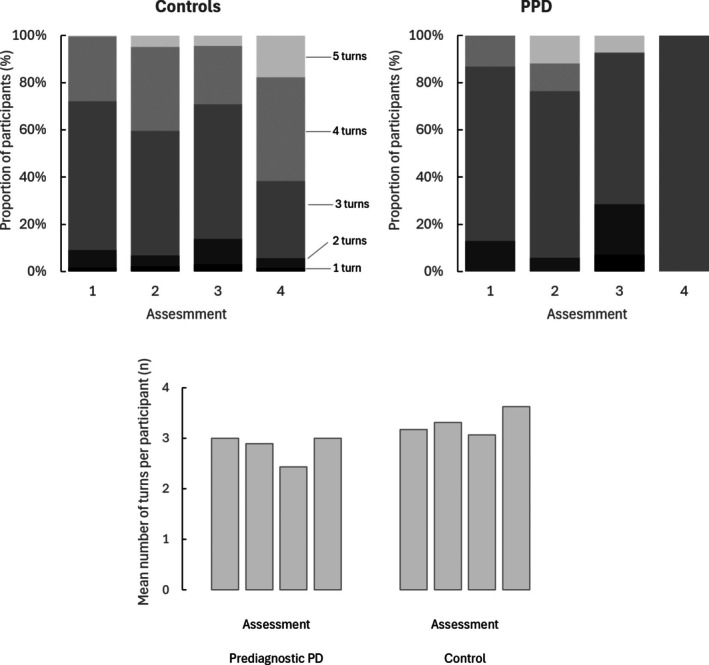
Illustrates the proportion of assessments during each visit as a function of the number of turns used to calculate turning characteristics for controls (left panel) and PPD after their first assessment (middle panel), as well as a comparison of the two cohorts' average number of turns in each visit.

A total of 933 participants, 51 to 85 years old, were included in the linear mixed model analysis, of an initial 1,051 participants whose turning was assessed. Reasons for exclusion included the absence of longitudinal assessments (n = 90), incomplete demographic data because of participants aborting the assessment (n = 17), and assessments that were incorrectly performed and had to be discarded (n = 3). An additional 6 participants were excluded because of a diagnosis of vascular PD (n = 3), or secondary PD (n = 3). Two participants were excluded as they were suspected of having PD but awaited further clarification, and both preferred not to proceed with PD diagnostics because of other health concerns. Nine controls were excluded from the survival analysis because they aborted the movement assessment before recording meaningful turns. Of the 924 participants included in the survival analysis, 23 were diagnosed with idiopathic PD during the observation period, with a mean of 5.3 years (σ = 3.6 years, range: 0.4–10.5) between their first turning assessment and diagnosis. The remaining 901 participants, who were not diagnosed with PD, had a mean time until censoring of 7.7 years (σ = 2.4 years, 2.0–11.0) after their initial turning assessment. The mean age of PPD was 5.1 years older than HC participants at the time of their first assessment (*p* < 0.001) (Table 1) (Figure A1).

**TABLE 1:** Demographic and Clinical Characteristics for Participants Included in the Linear Mixed Effects Models of Annual Change of Turning Performance (n = 933)

Characteristic	Control cohort (n = 910)	PPD (n = 23)
F, n (%)	485 (53.3)	3 (13.0)
Age, yr	66.1 (6.6)	71.6 (5.5)
BMI, kgm^−2^	26 (4)	26 (3)
Education, yr	14 (3)	15 (3)
MMSE, 0–30	28 (2)	28 (2)
CERAD, 0–82	84 (16)	81 (9)
BDI‐I score	6 (6)	6 (4)
Self‐reported depression, n (%)	69 (7.5)	1 (4.3)

Data presented in means and (standard deviation), unless otherwise indicated. Data are derived from first turning assessment of each participant.

BDI‐I = Beck Depression Inventory I; BMI = body mass index; CERAD Consortium to Establish a Registry for Alzheimer's Disease neuropsychological battery; F = female; MMSE = Mini‐Mental State Examination; PPD = prediagnostic Parkinson's disease cohort.

### Changes in Turning Measures over Time

**FIGURE A2:**
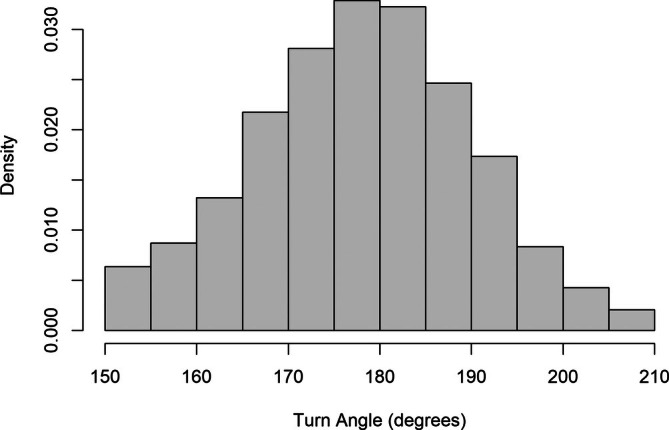
Illustrates the distribution of the angle of turns included in the final analysis.

Over time, participants tended to turn using a wider arc (smaller turn angle) and more slowly (Table 2). More specifically, linear mixed effects models of longitudinal turning assessment showed a significant (*p* < 0.05) effect of aging resulting in a wider arc, longer turn duration, and slower average and peak angular turning velocity. Although angular velocity slowed for the start and middle of the turn, angular velocity at the end of the turn did not change significantly over time. Participants who went on to receive PD diagnosis turned with a larger arc and slower peak angular turning velocity compared to controls at (modelled at age of diagnosis, 77.1 years). An age by clinical diagnosis status interaction indicated that turn angle showed a tendency to decrease more in PPD over time (before diagnosis) than those who did not receive the diagnosis later. When we ran the same models, but with only PPD, we confirmed significant reductions of both peak angular velocity and turn angle before diagnosis (*p* < 0.05), as mentioned in the method section, we refrained from drawing conclusions from the turn angle parameter. However, although all participants turned 180° in practice, the turn angle measured by the algorithm differed (Figure A2). Furthermore, female participants tended to turn with a wider arc, shorter duration, quicker average and starting angular velocities, and slower peak angular velocities compared to males.

**TABLE 2:** Yearly Change of Turning Measures Over 10 years

Predictor	Intercept	Sex (F)	PD status (Parkinson's)	Age (yearly change)	PD status by age interaction (PD, yearly change)
Turn angle (°)	177 (175–179)	**−1.6** ^**a**^ **(−2.1 to −1.1)**	**−6.4** ^**a**^ **(−9.6 to −3.1)**	**−0.20** ^**a**^ **(−0.26 to −0.15)**	**−0.84** **(−1.44 to −0.24)** ^a^, ^b^
Turn duration (s)	2.36 (2.32–2.41)	**−0.04** ^**a**^ **(−0.06 to −0.02)**	0.01 (−0.13 to 0.15)	**0.02** ^**a**^ **(0.01–0.02)**	−0.01 (−0.04 to 0.01)
Turn angular velocity (°s^−1^)					
Whole turn	78.7 (77.7–79.7)	**0.9** ^**a**^ **(0.2–1.7)**	−2.6 (−8.0 to 2.7)	**−0.68** ^**a**^ **(−0.86 to −0.51)**	0.24 (−0.73 to 1.21)
Start of turn	34.8 (33.6–36.1)	**2.6** ^**a**^ **(1.8–3.4)**	1.14 (−4.6 to 6.9)	**−0.49** ^**a**^ **(−0.56 to −0.42)**	0.68 (−0.35 to 1.71)
Middle of turn	127.2 (124.5–129.9)	−0.1 (−2.0 to 1.9)	−8.6 (−21.9 to 4.6)	**−0.96** ^**a**^ **(−1.20 to −0.73)**	−0.34 (−2.73 to 2.06)
End of turn	31.2 (30.4–32.0)	0.2 (−0.5 to 0.8)	−2.5 (−6.8 to 1.9)	−0.06 (−0.18 to 0.07)	−0.35 (−1.14 to 0.45)
Peak	186.6 (183.1–189.9)	**−4.0** ^**a**^ **(−5.9 to −2.1)**	**−23.7** ^**a**^ **(−36.7 to −10.7)**	**−1.49** ^**a**^ **(−1.83 to −1.15)**	−1.19 (−3.54 to 1.16)^a^, ^b^

Estimates are presented as the model coefficient (95% confidence intervals). Estimates are derived from a linear mixed effects model with sex, age, Parkinson's status, and age by clinical diagnosis status interaction entered as fixed effects, and random intercepts and slopes fitted for participants. Estimates are for a male either at age of diagnosis of PD for those who developed PD or, for controls, the mean age at diagnosis of participants who did develop PD (77 yr).

^a^ Bolded estimates are significantly different to 0 (*p* < 0.05).

^b^ Significant change over time (yearly change) in the PPD cohort (*p* < 0.05).

F = female; PD = Parkinson's disease.

### Survival Analysis of PD Diagnosis

Older age at baseline and male sex were significantly associated with a high hazard of PD, where a 1‐year increase of age was associated with a 15% higher hazard of developing PD, and being male was associated with a fourfold hazard of developing PD (Table 3). Baseline body mass index (mean = 26.4, σ = 4.2kg·m^−2^), global cognition (MMSE, mean = 28.5, σ = 1.4) and depressive symptoms (BDI, mean = 5.9, σ = 5.9) did not contribute significantly to the model (*p* > 0.30) and so were not included in the base model.

**TABLE 3:** Cox proportional hazard regression of the association between developing PD and baseline demographic and turning characteristics

Predictor	Baseline mean (SD)^a^	Model coefficients exp (β) (CI_95%_)	*p*
Intercept			
Sex (reference = M)^b^	M = 440 (48%)		
	F = 484 (52%)	0.199 (0.059–0.671)	**0.009**
Age (yr)^b^	66.0 (6.5)	1.149 (1.071–1.232)	**<0.001**
Turn angle (°)^c^	179 (10)	0.970 (0.930–1.012)	0.162
Turn duration (s)^c^	2.2 (0.4)	1.299 (0.466–3.621)	0.617
Turn angular velocity (°s^−1^)			
Whole turn^c^	84 (16)	0.987 (0.957–1.017)	0.395
Start of turn^c^	39 (14)	0.990 (0.955–1.026)	0.588
Middle of turn^c^	138 (34)	0.993 (0.979–1.007)	0.310
End of turn^c^	32 (8)	0.972 (0.920–1.026)	0.302
Peak angular velocity^c^	201 (42)	0.988 (0.976–1.000)	**0.045**

*Note:* Bold indicates significant *p*‐values.

^a^ Baseline turning measures are derived from a model corrected for age (centered at 66 yr, mean baseline age of the whole cohort) and sex (reference: M).

^b^ Coefficients for age and sex represent the base model, with both characteristics force entered the model.

^c^ Models include the base model: (age and sex) with the addition of individual turning measures.

CI = confidence interval; F = female; M = male; SD = standard deviation.

When added to the base model, a slower peak angular velocity was significantly associated with a greater hazard of developing PD. No other turning characteristic was significantly associated with the hazard of developing PD when added individually to the base model. Scaled Schoenfeld residuals were not correlated with time for model factors (*p* > 0.2), supporting the assumption of proportionality of hazards.

The up‐sampled XGBoost classification model, using baseline characteristics of age, sex, and peak angular velocity, identified 60% of PPD (sensitivity: 0.600) and 80.5% non‐PPD (specificity: 0.805), with an overall area under the curve (AUC) of 80.5%. The classification model resulted in a high negative predictive value (98.9%), but low positive predictive value (6.3%).

### When before PD Diagnosis Does Turning Change?

**FIGURE 1:**
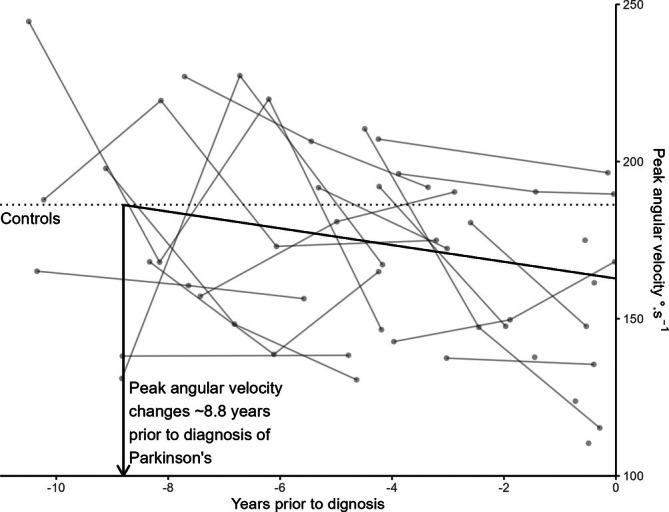
Illustrates change in peak angular velocity in n = 23 older adults prior to a diagnosis of PD (grey points and lines) compared to the mean of n = 910 older adults modelled at the average age of PD diagnosis (77.1 y, horizontal dotted line, controls). The sloped black line shows the mean modelled change of peak angular velocity (2.68 °· s^−1^ · y^−1^) in the PPD cohort. The solid black vertical line indicates that the mean lines of the PPD and controls intersect approximately 8.8 years prior to diagnosis.

We hypothesized that the latency of decline in turning measures before the diagnosis of PD is similar to that observed for gait characteristics, as described by Postuma et al.^30^ Peak angular velocity was analyzed as it was the only characteristic that both changed over time in the PD cohort and was associated with increased hazard of PD. Using data from Table 2, mean peak angular velocity of PPD was 162.9° · s^−1^ at time of diagnosis and of 186.6° · s^−1^ for controls modelled at 77.1 years (mean age of PD diagnosis). Peak angular velocity in PPD slowed by 2.68° · s^−1^ · y^−1^, suggesting turning measures of PPD started to deviate from controls (186.6° · s^−1^ to 162.9 ° · s^−1^) ÷ 2.68° · s^−1^ · y^−1^ ≈ 8.8 years before diagnosis (Figure 1).

## Discussion

This is, to our knowledge, the first study to longitudinally assess turning in older adults at risk of PD, with multiple follow‐ups. We found that peak angular velocity distinguishes PPD from controls up to 8.8 years before clinical diagnosis. Our results suggest that turning measures may aid in predicting the clinical PD diagnosis and enhance a panel of prediagnostic markers for identifying high‐risk individuals. Using a single wearable inertial measurement unit and validated algorithms, the approach used in this study is practical for large‐scale screening for PD.

### Change over Time

Participants in the PPD cohort started to turn with larger arcs and lower velocity than their counterparts who did not develop PD later. This finding is, at least indirectly, in agreement with recent work,^31^ which detected a decline in mean and peak angular velocity in participants with idiopathic RBD, a cohort associated with high risk of PD. The deterioration of peak angular velocity might be a sign of deterioration either in general speed of motion or in balance and postural stability. Both domains are very often affected in PD.^1^, ^9^ A decline in postural stability may subconsciously “incentivize” patients to turn slower and adopt a safer route using a larger arc. This suggestion is in line with findings showing peak angular velocity and number of steps during turning as an important marker for balance confidence in multiple sclerosis patients.^32^ This would mean that PD patients develop, already in the prediagnostic phase, strategies for safer and more stable turning.^33^

Independent of PD diagnosis, our findings show turning with longer duration, lower peak and average angular velocity, and wider arc with aging in participants who were not diagnosed with PD later. Along with lower angular velocity at the start and middle of turning, these changes with age could be described as physiological aging. Surprisingly, we did not find significant sex‐based differences, only tendencies were noted. To date, there is a lack of reliable normative wearable device data on turning measures from large cohorts of older adults, which limits the possibility of meaningfully comparing these findings to existing studies.

### Can Changes in Turning Predict PD Diagnosis?

To our knowledge, only a few cross‐sectional studies currently published are reporting about the potential of quantitative turning characteristics^9^; or even only using circular walking,^34^ which is relatively similar, yet considerably different from 180° turning. Our findings show, for the first time, a decline of peak angular velocity in PPD, years before the clinical diagnosis.

Peak angular velocity has been shown to be an effective metric for distinguishing between controls and individuals with mild postural deficits, even when gait remains fairly similar in both cohorts.^35^ As discussed above, it is possible that the deterioration in peak angular velocity is a result of a subtle deficit in balance, a present issue in most PD patients. Regardless of the patho‐mechanism behind the declined peak angular velocity, it appears to have a significant predictive value of PD. Furthermore, our classification model considering age, sex, and peak angular velocity performed reasonably well with an AUC of 80.5%. Although a higher sensitivity (0.6) of the model would be preferable, a specificity of 0.8 shows promise for the model to be relevant and avoid unnecessary follow‐ups or anxiety for participants unlikely to develop PD.

### Latency of Preclinical Changes in Turning

We originally hypothesized that, similar to gait, turning changes in PPD appear 4 to 5 years before diagnosis.^30^ Our findings show that, at time of diagnosis, the PPD cohort both turned with a slower peak angular velocity and longer arc compared to participants who were not subsequently diagnosed with PD. Moreover, we estimate that the deviation of turning measures in PPD from physiological aging start approximately 9 years before the diagnosis. According to these results, peak angular velocity may be among the earliest motor markers of PD, although care should be taken given the modest number of PPD limits the precision of our conclusions. These difficulties in turning can be considered pathological changes. The complexity of turning as a movement^12^ deems it likely that the decline of turning in PPD is multifactorial. For example, mobility deficits, rigidity, and bradykinesia have been reported to decline years before the clinical diagnosis,^36^ and they can all contribute to the decline of turning quality. Other factors such as eye‐to‐foot coordination,^37^ balance,^38^ and locomotion specific deficits could also play a rule, however, they are, to our knowledge, yet to be investigated in more detail in PPD. It would be of interest to explore further changes in turning in PPD, in subtypes of PD as well as other factors that may influence turning, such as lateralization of PD.

### A Panel of Motor Markers for PD?

As mentioned above, the promising findings shown here could contribute to a clinical panel for motor markers of PPD, based on available findings.^9^ This panel would include peak angular velocity (as presented here), along with voice and face akinesia—symptoms that show the earliest deviations from normal findings, emerging 9 years before diagnosis.^30^ These are followed by finger tapping (8 years),^30^ wrist acceleration (arm swing deterioration, at 7 years),^10^ mobility deficits (6 years), lower gait rhythm with increased variability and asymmetry (4.5 years),^11^ and finally, balance deficits, rigidity, and limb bradykinesia (4 years).^30^, ^39^ Most of these markers can be easily and affordably assessed using wearable digital devices.

### Limitations and Strengths, and Future Work

To our knowledge, this is the first prospective longitudinal study on turning measures in PPD. However, the work presented has some limitations. For instance, we only included 7 turning measures. Future work can investigate more turning characteristics (eg, as described by Rehman et al^40^), also in combination with gait and balance characteristics. Furthermore, the algorithm used for analysis of a lumbar wearable device data has limitations in terms of turning angle detection (provided in Figure A2). This is because of the challenge of reliably defining the start and end of a turn when a single wearable device is used on the lower back.^25^ Moreover, the limited number of PPD participants may affect precision and generalizability of the results. In addition, subtle changes in the prediagnostic stages of turning necessarily result in wide CIs when estimating how long before diagnosis changes in turning measures occur. Although our results align with previous studies, incorporating a larger sample of PPD along with more intricate nonlinear statistical models (currently unfeasible because of the moderate sample size) will produce more precise estimates.

**FIGURE A3:**
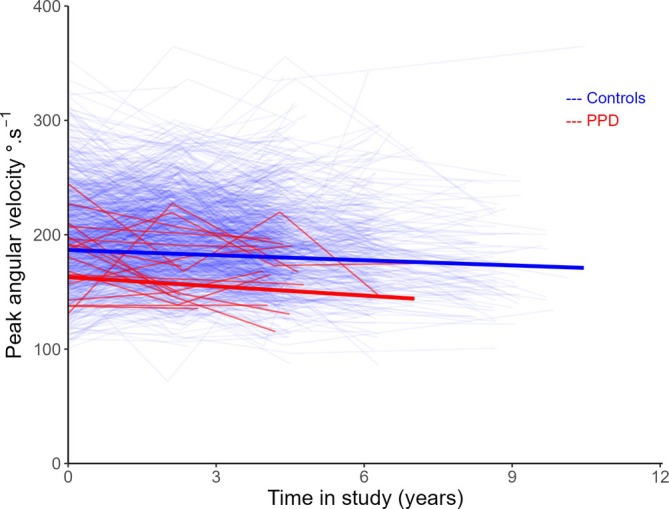
Illustrates trajectories of peak angular velocity of turning of n = 23 older adults prior to a diagnosis of PD (red) and n = 910 who were not diagnosed with PD (blue) over the years. Thin lines indicate individual participants, whilst thicker solid lines indicate unadjusted linear models fit data included in longitudinal analysis. [Color figure can be viewed at www.annalsofneurology.org]

Our findings indicate that quantitative, digital device‐based assessments of turning movements could be a valuable component in a diagnostic battery for identifying PPD or early subclinical motor signs. Future work should also focus on higher sensitivity and specificity of the machine learning model, which may serve as a step toward an efficient, clinically relevant screening of PD. Finally, although our findings provide valuable insights into the prediagnostic phase of PD, future work is warranted to further investigate the specificity of these changes in PD compared to other neurodegenerative diseases (Figure A3). Peak angular velocity in PPD slowed by 2.68° · s^−1^ · y^−1^, suggesting turning measures of PPD started to deviate from controls (186.6 ° · s^−1^ − 162.9 ° · s^−1^) ÷ 2.68 ° · s^−1^ · y^−1^ ≈ 8.8 years prior to diagnosis (Figure 1).

## Author Contributions

M.E. B.G., W.M., D.B., and C.H. contributed to the conception and design of the study; M.E., C.H., M.H., A.K.v.T., K.B., F.M., B.T., D.B., W.M., and B.G. contributed to the acquisition and analysis of data; M.E., B.G., and W.M. contributed to drafting the text or preparing the figures.

## Potential Conflict of Interest

Nothing to report.

## Data Availability

The data that support the findings of this study are available from the corresponding author on reasonable request.
